# Children’s Processing of Written Ironic Praise and Ironic Criticism: Evidence from Eye-Tracking Analyses

**DOI:** 10.3390/bs16071101

**Published:** 2026-07-02

**Authors:** Jiayi Zhong, Junsheng Liu

**Affiliations:** 1 School of Foreign Languages, Huazhong University of Science and Technology, Wuhan 430074, China; zhongjy@hust.edu.cn; 2 Shanghai Key Laboratory of Mental Health and Psychological Crisis Intervention, School of Psychology and Cognitive Science, East China Normal University, Shanghai 200062, China

**Keywords:** ironic praise, eye tracking, written irony, middle childhood, nonliteral language

## Abstract

This study investigated how children aged 7 to 11 years process and comprehend written irony with different emotional valences (ironic praise vs. ironic criticism) using eye-tracking technology. Participants read short stories containing literal praise, literal criticism, ironic praise, or ironic criticism while their eye movements were recorded. Results indicated that children showed significantly lower comprehension accuracy for ironic praise compared to ironic criticism, supporting the affective asymmetry hypothesis in irony processing. Eye-tracking data provided partial support for this asymmetry: regression-path durations—but not first-pass or total reading times—were longer for ironic utterances, particularly ironic praise, indicating greater effort during integrative rereading and reanalysis. Age-related differences were limited to regression-path duration rather than comprehension accuracy, suggesting selective developmental differences in online integration. These findings provide process-level evidence for children’s written irony comprehension and highlight the role of online integrative processes in figurative language processing.

## 1. Introduction

Interpreting other people’s intentions through verbal language represents a remarkable challenge for children. Much of what we say is ambiguous; the intended meaning of our statements cannot always be gleaned from the literal meanings of the words alone. One particularly challenging form of figurative language is counterfactual verbal irony, in which the intended meaning is opposite to the literal utterance (Katz & Lee, 1993). While children’s acquisition of spoken irony has been extensively studied, less is known about their online processing of written irony, which lacks prosodic or paralinguistic cues. Furthermore, the well-documented asymmetry between ironic criticism and ironic praise—where critical irony is understood more readily—has rarely been examined in real-time reading tasks among children. This study used eye tracking to examine these online processing patterns during irony comprehension in middle childhood.

### 1.1. Processing of Written Irony

Comprehending irony typically involves resolving a discrepancy between the literal meaning of a statement and the expectations derived from the context. Several theoretical accounts have been proposed. Grice’s (1975) conversational implicature theory regards irony as a violation of the Maxim of Quality, whereby speakers intentionally produce a literally false utterance and rely on listeners to infer the intended meaning. In contrast, Relevance Theory (Sperber & Wilson, 1986/1995) proposes that irony involves the echoic interpretation of a prior thought or expectation, together with an attitude toward it, rather than simply saying the opposite of what is meant. More recently, the Parallel Constraint Satisfaction Framework (PCSF; Pexman, 2008) has provided a useful account of this pattern by proposing that irony comprehension results from the simultaneous integration of multiple constraints, including contextual information, literal meaning, and speaker intention. According to this framework, children gradually acquire different strengths of association between these constraints through experience.

Counterfactual verbal irony is a form of irony in which the literal statement contradicts a factual expectation, for instance, saying, “*What a perfect day for a picnic!*” when it is raining. This contrasts with non-counterfactual irony, where literal and intended meaning diverge less dramatically. Written irony often relies on subtle contextual cues rather than overt prosody, making integration and inference more demanding. Eye-tracking studies with adults demonstrate that ironic statements are read more slowly, involve more regressions, and are comprehended less accurately than literal statements (Au-Yeung et al., 2015; Filik & Moxey, 2010; Olkoniemi et al., 2016), reflecting the cognitive effort required to reconcile literal content with context.

In developmental populations, children must detect discrepancies between literal meaning and context while simultaneously interpreting the speaker’s evaluative stance. Previous studies indicate that children may succeed at detecting ironic statements but struggle to integrate them fully, particularly in the absence of prosodic cues (Olkoniemi et al., 2023; Barich et al., 2025).

### 1.2. Ironic Praise and Ironic Criticism

Ironic statements can serve positive or negative social functions. Ironic criticism involves stating something positive literally to convey a negative evaluation (e.g., “This audience is very pleasant” after an audience that behaved disruptively), whereas ironic praise involves stating something negative literally to convey a positive evaluation (e.g., “Well, that was a terrible shot” to commend a good performance). Research consistently shows an affective asymmetry: children understand ironic criticism earlier and more accurately than ironic praise, likely because negative social feedback is more frequent and salient, creating a normative bias (Filippova & Astington, 2010; Hancock et al., 2000; Pexman et al., 2005). This asymmetry is likely due to greater exposure to critical irony in daily social interactions and the increased cognitive demands of resolving positive evaluations from negative phrasing. Ironic praise appears to require more advanced cognitive, linguistic, and social–pragmatic skills, including theory of mind and perspective taking.

### 1.3. Eye-Tracking Studies of Children’s Irony Processing

While children’s acquisition of verbal irony is well documented (Climie & Pexman, 2008; Köder & Falkum, 2021), fewer studies have examined online reading of written irony. Existing eye-tracking research suggests that although children around age 10 exhibit adult-like patterns in some measures, comprehension accuracy remains lower, and rereading behavior indicates additional cognitive effort (Olkoniemi et al., 2023; Barich et al., 2025). Specifically, longer regression-path durations and increased look-backs indicate that children engage in effortful integration to reconcile literal text with intended ironic meaning. However, prior studies have often focused on a single age group, limiting developmental inference, and have rarely examined differences between ironic praise and criticism in written text.

### 1.4. The Current Study

The present study investigates the developmental trajectory of written irony comprehension in children aged 7–11, with particular focus on affective asymmetry between ironic criticism and ironic praise. Among these accounts, the Parallel Constraint Satisfaction Framework (PCSF; Pexman, 2008) is particularly relevant to the present study because it predicts that irony comprehension depends on the simultaneous integration of multiple constraints, including contextual expectations, literal meaning, and speaker intentions. Importantly, the framework predicts that ironic praise should impose greater integrative demands than ironic criticism because positive evaluations expressed through negative wording constitute a less expected communicative pattern. The present study provides a developmental test of this prediction using eye-tracking measures of online reading.

We recruited 96 children in Shanghai across three age groups (7-, 9-, and 11-year-olds; 42 females). Based on the literature, we formulated the following hypotheses:

**H1.**  *Comprehension accuracy is higher for ironic criticism than for ironic praise across all age groups, demonstrating the affective asymmetry effect.*

**H2.**  *Eye-movement patterns (e.g., longer regression-path durations) indicate increased integrative effort for ironic utterances compared to literal ones, specifically for ironic praise compared to ironic criticism.*

**H3.**  *Developmental progression will be observed, with older children (11-year-olds) demonstrating comprehension accuracy and reading patterns closer to adult profiles than younger children (7-year-olds), especially for ironic praise.*

## 2. Materials and Methods

### 2.1. Participants

Ninety-six participants from an elementary school in Shanghai participated (42 females; mean age = 10.3 years), comprising three age groups: thirty-two 7-year-olds (mean age = 7.2; SD = 0.3), thirty-two 9-year-olds (mean age = 9.1; SD = 0.4), and thirty-two 11-year-olds (mean age = 11.3; SD = 0.5). For the power analysis, we assumed a medium effect size (Cohen’s d = 0.5), which is consistent with conventions in psychological research (Cohen, 2013) and with prior eye-tracking studies on irony processing (Filik & Moxey, 2010; Filik et al., 2014, 2017). Based on these parameters, a minimum of 32 participants per age group was required to achieve a statistical power of 80%, as recommended by Cohen (2013). All were native speakers of Mandarin Chinese (the language studied at the school) and had normal or corrected-to-normal vision. A parent/guardian of each participant gave written informed consent before the experiment.

### 2.2. Apparatus

Eye movements were recorded using EyeLink Portable Duo eye trackers (SR Research Ltd., Ottawa, On, Canada) at a 500 Hz sampling frequency. The stimuli were presented on a 13-inch laptop monitor, participants were seated 60 cm from the screen, and a refresh rate of 120 Hz and a resolution of 1024 × 768 pixels were used. A chin-and-forehead rest was used to stabilize the head of the participant.

### 2.3. Materials

Twelve story frameworks were created, each appearing in four text-type versions (literal criticism, ironic criticism, literal praise, and ironic praise). A Latin-square design was adopted to generate four experimental lists. Each participant viewed only one version of each framework and therefore completed 12 experimental items (3 items per text type), together with 24 filler items. Experimental and filler items were presented in a pseudo-randomized order. Each experimental item was followed by a memory question and a comprehension question. One point was awarded for each correct response, yielding a maximum score of 6 for each text type condition. The memory question ensured that participants attended to the contextual information necessary for irony interpretation. These materials were adapted from adult materials (Filik et al., 2017) by two experienced third-grade teachers to ensure age-appropriate vocabulary and sentence structures for children aged 7–11. A pre-test was conducted with 125 third-grade students (mean age = 8.9 years; 62 females). The initial stimulus pool consisted of 20 story frameworks (80 items). Items were excluded if (a) the mean difficulty rating exceeded 3.5 or (b) agreement on irony classification fell below 70% (Cohen’s κ < 0.70). Following this procedure, 12 story frameworks (48 experimental items) were retained. The four experimental conditions were matched on the basis of target utterance length (8 ± 1 Chinese characters) and mean difficulty ratings (all ps > 0.20).

We divided text into three regions of interest. The first is the context region (1), which provides the situational background and establishes expectations for the upcoming statement. It contains the critical contextual information that enables readers to anticipate the speaker’s intended meaning before encountering the target utterance. The spillover region (2) immediately follows the context region, whereas the critical region (3) contains the target utterance, whose intended meaning depends on the preceding context. An example text and questions are presented in Table 1.

### 2.4. Procedure

Participants were tested individually. Upon arrival, participants were informed that the experiment assessed reading. The specific nature of the experiment was explained to participants when the experiment was over. Before the reading task, the eye-tracking system was introduced to each participant, and the experimental procedure was explained. After completing a nine-point calibration procedure, participants started with 2 practice trials first to familiarize them with the experimental process. At the beginning of each trial, a fixation box appeared in the top-left quadrant of the screen. Once the participant fixated on this box, the box would disappear, and the text would be presented, with the beginning of the text appearing at the same location as the fixation box. After each story, two questions were presented, one at a time. Participants answered two yes/no questions after each story: (1) a text memory question (e.g., “Did Lu Hong say that Gao Zhen’s painting was good?”) and (2) a comprehension question assessing ironic intent (e.g., “Did Lu Hong actually think Gao Zhen’s painting was good?”). After the participant answered the second question, the next story was presented.

### 2.5. Data Analysis and Preprocessing

Fixations under 80 ms were incorporated into larger adjacent fixations within one character, and fixations under 40 ms that were not within three characters of another fixation were deleted, as were fixations over 1200 ms. Prior to analysis, trials where participants failed to read the sentence or there had been track loss were eliminated, accounting for 8.92% of the data. Three measures of reading behavior are reported: First-pass reading time (FRT) reflects initial lexical access and syntactic processing. A longer FRT would indicate early detection of incongruency. Regression-path duration (RPT)—the sum of all fixations from first entry into the region until the gaze exits to the right, including regressions to previous text—reflects early integrative difficulty, i.e., the reader’s attempt to reconcile the current utterance with prior context. The total reading time (TRT) indexes overall processing burden, including rereading after initial integration. Following previous irony eye-tracking studies (e.g., Olkoniemi et al., 2023), longer a RPT without a longer FRT suggests that difficulty arises during integration/reanalysis rather than during initial word recognition.

Participants with fewer than two correctly comprehended ironic items were excluded to ensure reliable measurement of irony processing. Participants must have demonstrated minimal comprehension ability to ensure data quality. Sensitivity analyses were conducted by re-running all primary mixed-effects models using the full sample, including the six participants excluded according to the predefined criterion. The overall pattern of results remained unchanged, indicating that the exclusion criterion did not materially affect the conclusions of the study.

The data were analyzed with linear mixed-effects models (LMMs) for continuous eye-movement measures (first-pass reading time [FRT], regression-path time [RPT], and total reading time [TRT]) and generalized linear mixed-effects models (GLMMs) with a binomial link function for accuracy data. Text type (literal criticism, literal praise, ironic praise, or ironic criticism) and age (7, 9, or 11) were treated as fixed effects, and their interaction was included in all models. Random intercepts for participants and items were included, and random effects were simplified as necessary to achieve model convergence. Unless otherwise specified, all results reported in the manuscript are based on these final simplified models. Models were compared based on χ^2^, the Akaike information criterion (AIC; Bozdogan, 1987), z-values, and *p*-values. The AIC is an estimator of the relative quality of statistical models for a given set of data and provides a means for model selection. The model providing the best fit to the data (lowest AIC value) was selected. Multiple comparisons were adjusted using Tukey’s Multiple Contrasts (part of the R “emmeans” package; Lenth, 2023) for post hoc testing. Post hoc pairwise comparisons were conducted using Tukey adjustments for multiple comparisons. The descriptive statistics presented in Table 2 were calculated from condition-level scores (maximum = 6 for each text type). In contrast, all inferential analyses were conducted on trial-level binary responses. Fixed-effect estimates are presented as regression coefficients (β), together with their standard errors (SEs), 95% confidence intervals (CIs), and z statistics. All statistical analyses were performed in the R language (Version 3.5.1; R Core Team, 2021) using the lme4 and emmeans packages (Lenth, 2023).

## 3. Results

### 3.1. Accuracy Performance

Descriptives for comprehension accuracy are presented in Table 2, and a summary of significant effects can be found in Table 3. Memory performance approached the ceiling across all conditions and age groups (M = 98%), indicating that participants successfully encoded the contextual information required for irony interpretation. Therefore, variation in total accuracy scores primarily reflected differences in comprehension rather than memory. There was a significant main effect of text type on comprehension accuracy. Post hoc Tukey comparisons reveal higher response accuracy for literal praise compared to literal criticism, ironic criticism, and ironic praise text types. Response accuracies were also significantly higher for literal criticism versus ironic praise, as well as for ironic criticism compared to literal criticism and ironic praise text types. Critically, supporting H1, accuracy was significantly higher for ironic criticism than for ironic praise. Furthermore, ironic praise was the least accurately comprehended text type.

### 3.2. Context Region of Eye Tracking

The context region provides the information necessary for readers to interpret the subsequent utterance as ironic or literal. The descriptive statistics for eye-tracking data are presented in Table 4. There were no significant effects of any factors on first-pass reading times in the context region. There were no other significant interactions or main effects among our factors across the remaining eye measures. Given that there were no interaction effects between text type and age in this region, our findings indicate that the effects of text type on first pass, regression path, and total reading times do not vary significantly between children of different age groups in the context region; children appear to have inspected the context region for ironic texts in the same way they did for literal texts (see Supplementary Materials for full model summaries).

### 3.3. Critical Region of Eye Tracking

The critical region is the segment of text that contains the ironic/literal utterance and follows from the context region.

*First pass.* No significant main effects or interactions were observed for first-pass duration.

**Figure 1:**
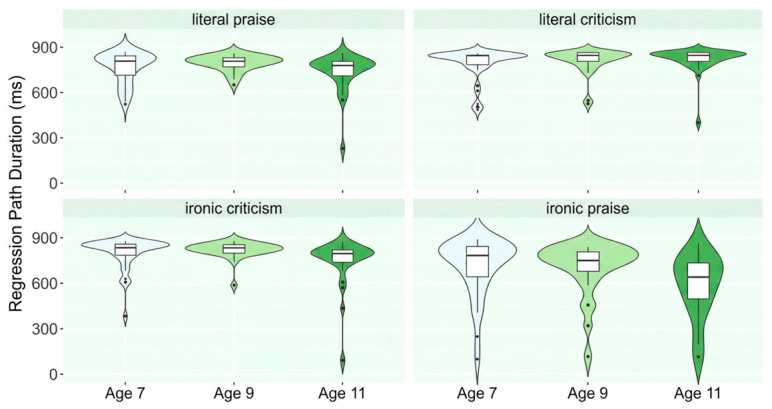
Violin plots showing regression-path reading times (simplified log10-transformed model) for text type and age group.

*Regression path.* There were no statistically significant interactions between any factors and regression-path duration in the critical region. However, there was a significant difference when comparing the base model to a text-type model (see Figure 1 and Table 5 for details). Post hoc comparisons showed that participants had longer regression-path durations for ironic praise remarks than they did for literal praise, ironic criticism, and literal criticism utterances. Participants also had longer regression-path durations for ironic criticism than they did for literal praise interactions and literal criticism. Compared to literal praise, participants had shorter regression-path durations for literal criticism interactions. There was no significant difference in regression path between literal praise and ironic criticism.

There was also a significant interaction effect between text type and age. Resolving age-by-text type interaction revealed significant differences in regression-path reading time for literal praise, with longer times for 9-year-olds compared to 11-year-olds. We found a similar difference for ironic criticism between 9-year-olds and 11-year-olds, with longer times for 9-year-olds. The regression-path reading time in the critical region was also significantly different for ironic praise interactions when comparing 9-year-olds and 11-year-olds, (9-year-olds > 11-year-olds) as well as 11-year-olds and 7-year-olds (7-year-olds > 11-year-olds). There were no other significant interaction effects. The observed interaction suggested that the processing difficulty associated with ironic praise (relative to other types) was more pronounced in younger children. Some conditions exhibited relatively large standard deviations, likely reflecting individual differences in reading behavior, consistent with developmental variability in irony comprehension (see Supplementary Materials for full model summaries).

*Total reading time*. There were no statistically significant interactions between any of our factors and total reading time in the critical region.

## 4. Discussion

We explored how children developmentally process and comprehend different types of written irony by employing the eye-tracking technique.

### 4.1. Accuracy

Our data generally showed that participants were more accurate when identifying literal criticism, literal praise, and ironic criticism than ironic praise. These findings align with previous research on irony comprehension in English-speaking children aged 7 to 11 (Filippova & Astington, 2010). In particular, ironic praise proved to be the most challenging for children to interpret. This difficulty can be partly attributed to the asymmetry of affect, which posits that ironic criticism is processed more readily than ironic praise (Hancock et al., 2000; Harris & Pexman, 2003; Cao et al., 2024). Filippova and Astington (2010) similarly reported that ironic criticism was easier to comprehend than ironic praise. Moreover, we observed that ironic criticism was not only easier to understand than ironic praise but also easier to understand than literal criticism statements. While this pattern is consistent with previous research suggesting that negative social information is generally more salient and easier to process than positive information (Baumeister et al., 2001; Rozin & Royzman, 2001), alternative explanations cannot be entirely ruled out. Because the present study did not independently manipulate or norm factors such as contextual support or stimulus naturalness, these characteristics may also have contributed to the observed advantage for ironic criticism. Future research should examine these possibilities more directly.

Contrary to H3, developmental effects were selective rather than global. Although age moderated regression-path duration, no reliable developmental progression was observed in comprehension accuracy, suggesting only limited developmental differences in online integration. Therefore, conclusions regarding developmental progression should be interpreted cautiously.

### 4.2. Eye Tracking

Our eye-tracking data provided partial support for H2. Although we did not observe the predicted overall slowdown in first-pass or total reading times for ironic versus literal utterances, the regression-path duration—an index of effortful integration—was significantly longer for ironic praise compared to all other text types and for ironic criticism compared to literal praise and literal criticism. This dissociation between intact first-pass times and prolonged regression-path durations suggests that the primary processing difficulty for written irony, particularly ironic praise, may lie more in the effortful reanalysis and integration of the nonliteral meaning with the context rather than in initial lexical access. However, the absence of first-pass effects should be interpreted cautiously, as null effects do not provide direct evidence of preserved early-stage processing. Thus, the evidence points to a localized integrative bottleneck rather than a global slowdown in reading.

Specifically, in the critical region, children exhibited longer regression-path durations for ironic utterances than for literal ones, indicating that both ironic praise and ironic criticism required additional effort to integrate the target utterance with the preceding context, consistent with classic theories of irony comprehension (Grice, 1975). Furthermore, the eye-tracking data paralleled the comprehension results: ironic praise elicited longer regression-path durations than ironic criticism, indicating that its lower comprehension accuracy was accompanied by greater online integrative effort rather than merely poorer final performance. This finding suggests that children experienced greater difficulty integrating the intended meaning of ironic praise with the preceding context.

Although previous eye-tracking studies have examined children’s irony processing (e.g., Olkoniemi et al., 2023; Barich et al., 2025), methodological differences limit direct comparison. Extending this literature, the present study is the first to directly compare ironic praise and ironic criticism using eye tracking and found that younger children showed disproportionately longer regression-path durations for ironic praise.

Taken together, the present findings suggest that children’s difficulty with written irony is best characterized as a localized integrative cost during rereading and reanalysis rather than as a broad disruption across all stages of processing. Children may detect the mismatch between literal meaning and context relatively quickly but require additional effort to resolve this mismatch in a coherent ironic interpretation, particularly for ironic praise.

### 4.3. Theoretical Implications

Our findings, particularly the dissociation between the robust accuracy asymmetry (H1) and the more selective eye-movement evidence (H2), have implications for theories of irony processing. While comprehension accuracy consistently favored ironic criticism over ironic praise, online processing differences were primarily reflected in regression-path duration, suggesting that processing outcomes and processing time course are only partially aligned. This pattern is broadly consistent with the Parallel Constraint Satisfaction Framework (Pexman, 2008), which proposes that multiple pragmatic constraints are simultaneously activated and integrated during interpretation. One possible explanation is that ironic praise involves weaker or less readily available pragmatic constraints, thereby requiring additional integrative processing during rereading, whereas ironic criticism may benefit from stronger contextual support. Although the present findings cannot directly test the mechanisms proposed by the framework, they are compatible with its account of irony comprehension and suggest that it may provide a useful perspective for understanding children’s online processing of written irony.

Beyond theoretical implications, the present findings have potential educational relevance. The greater difficulty associated with ironic praise suggests that positively intended ironic remarks may be particularly vulnerable to misunderstanding during middle childhood. Therefore, providing clearer contextual cues may support children’s pragmatic language development (Olkoniemi et al., 2025).

### 4.4. Limitations and Future Directions

One limitation of the present study is that, although we identified a localized integrative cost for ironic praise, it remains unclear how this processing difficulty translates into children’s emotional and social interpretations beyond basic comprehension accuracy. Future studies should combine eye tracking with measures of perceived speaker intent, humor, or emotional impact to determine how these interpretations evolve during irony comprehension (Filik et al., 2017), particularly for ironic praise and ironic criticism. In addition, although the four conditions were derived from the same story frameworks to minimize semantic variation, objective stimulus norms such as lexical frequency, emotional intensity, contextual predictability, and naturalness ratings were not independently collected. Future research should incorporate more extensive stimulus norming procedures to further reduce potential confounding influences. Another limitation is that the present study focused primarily on developmental differences at the group level and did not consider individual socio-emotional characteristics that may influence irony comprehension. According to the Parallel Constraint Satisfaction Framework (Pexman, 2008), exposure to irony in social interactions contributes to the development of irony understanding. Therefore, future studies should investigate how individual differences, such as with respect to shyness (Mewhort-Buist & Nilsen, 2017) or theory of mind, shape children’s interpretation of different forms of irony. Including adult control groups would also provide a useful benchmark for mature irony processing and help clarify developmental changes across childhood. Finally, the present study was conducted with Mandarin-speaking children in Shanghai, China. Cultural differences in the frequency and use of irony in everyday communication may influence the development of irony comprehension. Future cross-cultural research is therefore needed to examine the generalizability of the present findings.

## 5. Conclusions

This study provides novel eye-tracking evidence for children’s processing of written irony, highlighting a robust asymmetry whereby ironic praise poses greater integrative difficulty than ironic criticism. Although age-related differences were selective rather than pervasive, the findings suggest that the online integration of pragmatic meaning, particularly for positive-valence irony, remains effortful throughout middle childhood. By combining offline comprehension and online eye-movement measures, the present study provides process-level evidence for children’s written irony comprehension and demonstrates that the Parallel Constraint Satisfaction Framework offers a useful account of how competing pragmatic constraints are resolved during irony processing.

## Figures and Tables

**Table 1:** Examples of experimental texts and text memory and inference questions (translated from Mandarin).

Text Type	Region	Example Sentence
Literal criticism	Context region	Gao Zhen and Lu Hong attend art classes. Gao Zhen tells Lu Hong she is a good artist. In the class, they paint a picture of a rose. Gao Zhen’s painting is ugly.
	Spillover region Critical region	Lu Hong says, “Woah, you are a terrible artist.”
Literal praise	Context region	Gao Zhen and Lu Hong attend art classes. Gao Zhen tells Lu Hong she is a good artist. In the class, they paint a picture of a rose. Gao Zhen’s painting is pretty.
	Spillover region Critical region	Lu Hong says, “Woah, you are a terrific artist.”
Ironic criticism	Context region	Gao Zhen and Lu Hong attend art classes. Gao Zhen tells Lu Hong she is a bad artist. In the class, they paint a picture of a rose. Gao Zhen’s painting is ugly.
	Spillover region Critical region	Lu Hong says, “Woah, you are a terrific artist.”
Ironic praise	Context region	Gao Zhen and Lu Hong attend art classes. Gao Zhen tells Lu Hong she is a good artist. In the class, they paint a picture of a rose. Gao Zhen’s painting is pretty.
	Spillover region Critical region	Lu Hong says, “Woah, you are a terrible artist.”
Example comprehension questions	Memory	Did Lu Hong say, ‘You are a terrific artist’?
	Inference	Did Lu Hong think Gao Zhen’s painting was actually good?

**Table 2:** Comprehension accuracy scores for comprehension questions following four text types for children.

Age Group Text Type	Accuracy Mean (% Correct)			SD			Accuracy Range		
	7	9	11	7	9	11	7	9	11
Literal criticism	4.97 (83%)	5.10 (85%)	5.38 (90%)	0.48	0.41	0.28	3–6	3–6	3–6
Ironic criticism	5.80 (97%)	5.84 (97%)	5.92 (99%)	1.74	1.53	1.21	4–6	5–6	5–6
Literal praise	5.88 (98%)	5.90 (98%)	5.94 (99%)	0.34	0.31	0.24	5–6	5–6	5–6
Ironic praise	3.87 (65%)	4.44 (74%)	4.92 (82%)	1.09	1.01	0.86	1–6	2–6	4–6

Note. The maximum possible comprehension accuracy score for each text type (literal and ironic) was 6. One-sample *t* tests confirmed that children of all age groups performed significantly above chance on all text types. SD, standard deviation.

**Table 3:** GLMER model and post hoc results for irony comprehension accuracy. *P*-values were adjusted using the Tukey HSD method.

**Models**			**AIC**	**Chisq**	***p* Value**
Accuracy ~ 1 + (1|Subject) + (1|Item),			56,246		
Accuracy ~ Text Type + (1|Subject) + (1|Item),			40,017	16,229	<0.0001
Accuracy ~ Text Type * Age + (1| Subject) + (1|Item),			39,814	202.26	<0.0001
**Post hoc comparisons—Text Type**	**ß**	**SE**	**z-ratio**	**95%CI**	***p* Value**
Literal criticism–literal praise	−1.85	0.58	−3.21	[−2.99, −0.71]	0.0013
Literal criticism–ironic praise	0.76	0.27	2.85	[0.23, 1.29]	0.0044
Literal criticism–ironic criticism	−1.40	0.47	−2.97	[−2.32, −0.48]	0.0030
Literal praise–ironic praise	2.61	0.63	4.12	[1.37, 3.85]	<0.0001
Literal praise–ironic criticism	0.45	0.20	2.21	[0.06, 0.84]	0.027
Ironic praise–ironic criticism	−2.16	0.59	−3.65	[−3.31, −1.01]	<0.0001

Note. The asterisk (*) between variable names denotes an interaction effect.

**Table 4:** Descriptive statistics for eye-movement measures across three regions.

(ms)	Region	FRT			RPT			TRT		
Text Type	Age	7	9	11	7	9	11	7	9	11
Literal Criticism *M* (*SD*)	Context region	312 (169)	295 (155)	285 (140)	293 (186)	285 (162)	270 (145)	327 (224)	310 (198)	298 (175)
	Spillover region	288 (154)	275 (1380)	265 (125)	310 (172)	295 (150)	285 (132)	350 (210)	330 (185)	315 (165)
	Critical region	335 (188)	315 (165)	305 (150)	365 (210)	340 (180)	325 (160)	420 (255)	395 (225)	375 (200)
Ironic Criticism *M* (*SD*)	Context region	452 (313)	420 (285)	395 (260)	441 (350)	405 (310)	385 (275)	933 (934)	850 (870)	780 (820)
	Spillover region	480 (330)	445 (300)	420 (275)	520 (365)	480 (330)	450 (295)	1105 (950)	980 (900)	900 (850)
	Critical region	550 (350)	500 (320)	470 (300)	620 (400)	560 (360)	520 (330)	1250 (1100)	1100 (1050)	1020 (980)
Literal Praise *M* (*SD*)	Context region	305 (162)	290 (150)	280 (135)	290 (180)	280 (155)	265 (140)	320 (218)	305 (190)	295 (170)
	Spillover region	282 (148)	270 (132)	260 (120)	305 (167)	290 (145)	280 (128)	345 (205)	325 (180)	310 (160)
	Critical region	330 (182)	310 (160)	300 (145)	358 (205)	335 (175)	320 (155)	415 (250)	390 (220)	370 (195)
Ironic Praise *M* (*SD*)	Context region	430 (295)	400 (270)	378 (245)	418 (325)	388 (295)	368 (260)	880 (890)	800 (830)	740 (785)
	Spillover region	455 (305)	425 (285)	400 (260)	495 (340)	455 (315)	428 (280)	1050 (920)	930 (875)	860 (825)
	Critical region	520 (335)	480 (305)	450 (285)	585 (385)	530 (345)	495 (315)	1180 (1050)	1040 (1000)	960 (935)

**Table 5:** LMER model and post hoc results for regression-path duration. *P*-values were adjusted using Tukey’s HSD method.

**Model**	**AIC**	**Chisq**	***p* Value**
Regression path ~ 1 + (1|Subject) + (1| Item)	10,278		
Regression path ~ Text Type + (1|Subject) + (1|Item)	9636	848.81	<0.0001
Regression path ~ Text Type * Age + (1| Subject) + (1|Item)	9327	124.92	<0.0001
**Post hoc comparisons—Text Type**	**ß**	**z-ratio**	***p* Value**
Literal praise–ironic criticism	−0.013	−35.97	0.1397
Literal praise–literal criticism	0.231	−23.46	<0.0001
Ironic criticism–literal criticism	0.145	−37.85	**<0.0001**
Ironic praise–literal praise	0.031	−36.95	**<0.0001**
Ironic praise–ironic criticism	0.029	−55.81	0.0001
Ironic praise–literal criticism	0.163	−16.94	**<0.0001**
Post hoc comparisons—Age * Text Type	**ß**	**z-ratio**	***p* Value**
**Text Type = literal criticism**			
9-year-old–11-year-old	−0.008	−0.33	0.9398
9-year-old–7-year-old	0.018	0.75	0.7289
11-year-old–7-year-old	0.026	1.13	0.4898
**Text Type = literal praise**			
9-year-old–11-year-old	0.061	2.44	**0.0383**
9-year-old–7-year-old	0.023	0.97	0.5901
11-year-old–7-year-old	−0.037	−1.60	0.2447
**Text Type = ironic criticism**			
9-year-old–11-year-old	0.072	2.91	**0.01**
9-year-old–7-year-old	0.023	0.96	0.6017
11-year-old–7-year-old	−0.049	−2.12	0.0858
**Text Type = ironic praise**			
9-year-old–11-year-old	0.126	5.08	**<0.0001**
9-year-old–7-year-old	−0.007	−0.31	0.9459
11-year-old–7-year-old	−0.134	−5.75	**<0.0001**

Note. The asterisk (*) between variable names denotes an interaction effect.

## Data Availability

The original data presented in the study are openly available in OSF at https://osf.io/28qyf/overview (accessed on 25 June 2026).

## Supplementary Materials

The following supporting information can be downloaded at: https://www.mdpi.com/article/10.3390/bs16071101/s1. Table S1: Simplified log10 transformed model for accuracy; Table S2: Simplified log10 transformed model for first pass reading time on the context region; Table S3: Simplified log10 transformed model for regression path reading time on the context region; Table S4: Simplified log10 transformed model for total reading time on the context region; Table S5: Maximal log10 transformed model for first pass reading time on the critical region; Table S6: Simplified log10 transformed model for regression path reading time on the critical region; Table S7: Maximal log10 transformed model for total reading time on the critical region. Appendix S1: Experimental Stimuli; Appendix S2: Pretest Results; Appendix S3: Timeflow of a trial (how screen switched).
