# Supplementary material for: Children’s Processing of Written Ironic Praise and Ironic Criticism: Evidence from Eye-Tracking Analyses
Source: Behav Sci (Basel). 2026 Jul 2;16(7):1101. doi: 10.3390/bs16071101 (PMC13405963; doi:10.3390/bs16071101)
Supplement: Supplementary file 1 [file behavsci-16-01101-s001.zip › behavsci-4311182-supplementary1.pdf]

Supplementary Material

Table S1

Simplified log10 transformed model for accuracy.

a. Random effects				
Groups	Name	Variance	SD	Correlation
Participant	(Intercept)	0.001	0.032	-
Item	(Intercept)	0.001	0.021	-
Residual		0.008	0.076	-

b. Fixed effects estimates					
Fixed effect	*B*	SE	*df*	*t*	*p*
**Intercept**	**0.258**	**0.010**	**14.67**	**23.63**	**< 0.001**
**Text Type**	**-0.036**	**0.002**	**667.16**	**-12.37**	**< 0.001**
Age	-0.000	<0.001	52.52	1.69	0.118
**Text Type x Age**	**-0.012**	**0.003**	**665.76**	**-3.59**	**<0.001**

Note. Model was simplified as the maximal model design proposed by Barr et al. (2013) resulted in a singular boundary.

Table S2

Simplified log10 transformed model for first pass reading time on the context region.

a. Random effects

Groups	Name	Variance	SD	Correlation
Participant	(Intercept)	0.008	0.087	-
Item	(Intercept)	0.007	0.082	-
Residual		0.066	0.258	-

b. Fixed effects estimates

Fixed effect	*B*	SE	*df*	*t*	*p*
**Intercept**	**2.439**	**0.019**	**65.075**	**130.534**	**<0.001**
Text Type	0.002	0.006	1878.708	0.358	0.642
Accuracy	-0.024	0.014	53.583	-1.876	0.054
Age	0.018	0.016	53.638	1.226	0.231
Text Type x Accuracy	-0.005	0.006	1883.532	-1.456	0.142
Text Type x Age	0.002	0.005	1876.259	0.098	0.946
Accuracy x Age	0.008	0.014	53.687	0.679	0.515
Text Type x Accuracy x Age	0.007	0.006	1882.302	1.228	0.217

Note. Model was simplified as the maximal model design proposed by Barr et al. (2013) resulted in a singular boundary or failed to converge.

Table S3

Simplified log10 transformed model for regression path reading time on the context region.

a. Random effects

Groups	Name	Variance	SD	Correlation
Participant	(Intercept)	0.021	0.144	-
Item	(Intercept)	0.018	0.133	-
Residual		0.096	0.312	-

b. Fixed effects estimates

Fixed effect	*B*	SE	*df*	*t*	*p*
**Intercept**	**2.824**	**0.030**	**70.064**	**94.470**	**<0.001**
Text Type	-0.000	0.007	1876.216	-0.048	0.962
Accuracy	-0.026	0.021	53.810	-1.241	0.220
Age	0.022	0.026	49.469	1.483	0.136
Text Type x Accuracy	0.005	0.008	1879.238	0.702	0.483
Text Type x Age	-0.007	0.007	1877.159	-0.944	0.345
Accuracy x Age	0.032	0.021	53.809	1.493	0.141
Text Type x Accuracyx Age	-0.001	0.008	1878.315	-0.139	0.890

Note. Model was simplified as the maximal model design proposed by Barr et al. (2013) resulted in a singular boundary or failed to converge.

Table S4

Simplified log10 transformed model for total reading time on the context region.

a. Random effects				
Groups	Name	Variance	SD	Correlation
Participant	(Intercept)	0.016	0.125	-
Item	(Intercept)	0.009	0.092	-
Residual		0.060	0.246	-

b. Fixed effects estimates					
Fixed effect	*B*	SE	*df*	*t*	*p*
**Intercept**	**2.675**	**0.023**	**77.892**	**116.156**	**<0.001**
Text Type	0.005	0.005	2002.340	0.893	0.372
Accuracy	-0.025	0.018	53.872	-1.365	0.178
Age	0.001	0.001	53.793	0.741	0.462
Text Type x Accuracy	-0.005	0.006	2005.714	-0.893	0.372
Text Type x Age	-0.001	0.005	2002.534	-0.261	0.794
Accuracy x Age	0.000	0.001	53.793	0.049	0.961
Text Type x Accuracy x Age	0.001	0.006	2007.183	0.180	0.857

Note. Model was simplified as the maximal model design proposed by Barr et al. (2013) resulted in a singular boundary or failed to converge.

Table S5

Maximal log10 transformed model for first pass reading time on the critical region.

a. Random effects

Groups	Name	Variance	SD	Correlation
Participant	(Intercept)	0.008	0.094	-
Participant	Text Type (ironic)	0.001	0.016	0.09
Item	(Intercept)	0.011	0.126	-
Residual		0.055	0.232	-

b. Fixed effects estimates

Fixed effect	*B*	SE	*df*	*t*	*p*
**Intercept**	**2.566**	**0.024**	**57.060**	**105.761**	**<0.001**
Text Type	-0.001	0.005	52.994	-0.228	0.820
Accuracy	0.004	0.015	54.063	0.279	0.782
Age	0.002	0.001	53.997	1.604	0.115
Text Type x Accuracy	-0.005	0.005	53.511	-0.880	0.383
Text Type x Age	-0.003	0.006	52.993	-0.618	0.539
Accuracy x Age	-0.003	0.014	54.062	-0.247	0.805
Text Type x Accuracy x Age	0.002	0.009	53.411	0.339	0.736

Note. Model reported used the maximal model proposed by Barr et al. (2013).

Table S6

Simplified log10 transformed model for regression path reading time on the critical region.

a. Random effects

Groups	Name	Variance	SD	Correlation
Participant	(Intercept)	0.016	0.125	-
Item	(Intercept)	0.016	0.113	-
Residual		0.072	0.265	-

b. Fixed effects estimates

Fixed effect	*B*	SE	*df*	*t*	*p*
**Intercept**	**2.789**	**0.025**	**71.776**	**109.688**	**<0.001**
**Text Type**	**0.017**	**0.008**	**2023.913**	**2.884**	**0.004**
Accuracy	0.000	<0.001	665.23	-0.30	0.761
Age	0.000	0.002	54.056	0.151	0.880
Text Type x Accuracy	-0.003	0.006	2025.618	-0.487	0.626
**Text Type x Age**	**0.000**	**<0.001**	**665.92**	**2.39**	**0.018**
Accuracy x Age	0.024	0.018	54.050	1.326	0.191
Text Type x Accuracy x Age	-0.003	0.006	2029.983	-0.474	0.636

Note. Model was simplified as the maximal model design proposed by Barr et al. (2013) resulted in a singular boundary or failed to converge.

Table S7

Maximal log10 transformed model for total reading time on the critical region.

a. Random effects

Groups	Name	Variance	SD	Correlation
Participant	(Intercept)	0.009	0.097	-
Participant	Text Type (Ironic)	0.000	0.019	-0.08
Item	(Intercept)	0.015	0.124	-
Residual		0.045	0.211	-

b. Fixed effects estimates

Fixed effect	*B*	SE	*df*	*t*	*p*
**Intercept**	**2.754**	**0.025**	**57.353**	**110.526**	**<0.001**
Text Type	0.001	0.001	54.006	0.519	0.606
Accuracy	-0.022	0.014	54.025	-1.560	0.119
Age	0.000	0.001	53.078	0.455	0.651
Text Type x Accuracy	-0.008	0.007	53.212	-1.343	0.181
Text Type x Age	-0.001	0.005	53.051	-0.252	0.794
Accuracy x Age	0.015	0.017	54.022	1.190	0.238
Text Type x Accuracy x Age	-0.004	0.006	53.178	-0.377	0.725

Note. Model reported used the maximal model proposed by Barr et al. (2013).
