# Supplementary material for: Children’s Processing of Written Ironic Praise and Ironic Criticism: Evidence from Eye-Tracking Analyses
Source: Behav Sci (Basel). 2026 Jul 2;16(7):1101. doi: 10.3390/bs16071101 (PMC13405963; doi:10.3390/bs16071101)

Supplementary Material

Appendix S1. Experimental Stimuli

This appendix provides the experimental story frameworks used in the study. Each framework was instantiated in four conditions: Literal Criticism (LC), Literal Praise (LP), Ironic Criticism (IC), and Ironic Praise (IP). For each framework, the original Chinese version, an English translatio are provided.

Framework 1 – Art Class

LP (Literal Praise)

Chinese: 高珍和路宏一起上画画班，高珍告诉路宏自己画的很好。在班上高珍画了一支玫瑰花，画的非常好。路宏说：“你真是画的太棒了”。

English: Gao Zhen and Lu Hong attended art class together. Gao Zhen told Lu Hong that she was a good artist. In class, Gao Zhen painted a rose, and it was beautiful. Lu Hong said, “You are such a great artist!”

IP (Ironic Praise)

Chinese: 高珍和路宏一起上画画班，高珍告诉路宏自己画的很烂。在班上高珍画了一支玫瑰花，画的非常好。路宏说：“你真是画的太烂了”。

English: Gao Zhen and Lu Hong attended art class together. Gao Zhen told Lu Hong that she was a terrible artist. In class, Gao Zhen painted a rose, and it was beautiful. Lu Hong said, “You are such a terrible artist!”

LC (Literal Criticism)

Chinese: 高珍和路宏一起上画画班，高珍告诉路宏自己画的很烂。在班上高珍画了一支玫瑰花，画的非常丑。路宏说：“你真是画的太烂了”。

English: Gao Zhen and Lu Hong attended art class together. Gao Zhen told Lu Hong that she was a terrible artist. In class, Gao Zhen painted a rose, and it was very ugly. Lu Hong said, “You are such a terrible artist!”

IC (Ironic Criticism)

Chinese: 高珍和路宏一起上画画班，高珍告诉路宏自己画的很好。在班上高珍画了一支玫瑰花，画的非常丑。路宏说：“你真是画的太棒了”。

English: Gao Zhen and Lu Hong attended art class together. Gao Zhen told Lu Hong that she was a good artist. In class, Gao Zhen painted a rose, and it was very ugly. Lu Hong said, “You are such a great artist!”

Framework 2 – Video Game

LP Chinese: 卢心告诉姜强自己玩游戏玩的很好。他俩一起玩电子游戏时卢心的游戏人物一直活到了游戏最后一关。姜强说，“你真的玩的好厉害啊！”

English: Lu Xin told Jiang Qiang that she was very good at playing games. When they played the video game together, Lu Xin’s character survived all the way to the final level. Jiang Qiang said, “You are really good at this!”

IP Chinese: 卢心告诉姜强自己玩游戏玩的很烂。他俩一起玩电子游戏时卢心的游戏人物一直活到了游戏最后一关。姜强说，“你真的玩的好烂啊！”

English: Lu Xin told Jiang Qiang that she was terrible at playing games. When they played the video game together, Lu Xin’s character survived all the way to the final level. Jiang Qiang said, “You are really terrible at this!”

LC Chinese: 卢心告诉姜强自己玩游戏玩的很烂。他俩一起玩电子游戏时卢心的游戏人物没活过第一关。姜强说，“你真的玩的好烂啊！”

English: Lu Xin told Jiang Qiang that she was terrible at playing games. When they played the video game together, Lu Xin’s character did not survive the first level. Jiang Qiang said, “You are really terrible at this!”

IC Chinese: 卢心告诉姜强自己玩游戏玩的很好。他俩一起玩电子游戏时卢心的游戏人物没活过第一关。姜强说，“你真的玩的好厉害啊！”

English: Lu Xin told Jiang Qiang that she was very good at playing games. When they played the video game together, Lu Xin’s character did not survive the first level. Jiang Qiang said, “You are really good at this!”

Framework 3 – Cooking

LP Chinese: 喻蓝说自己做的菜非常好吃。有天陈向来喻蓝家做客，尝到了喻蓝做的菜，味道非常好。陈向说：“你做的菜可真好吃啊。”

English: Yu Lan said that her cooking was very delicious. One day, Chen Xiang visited Yu Lan’s home and tasted her cooking. The food was excellent. Chen Xiang said, “Your cooking is really delicious!”

IP Chinese: 喻蓝说自己做的菜不太好吃。有天陈向来喻蓝家做客，尝到了喻蓝做的菜，味道非常好。陈向说：“你做的菜可不太好吃啊。”

English: Yu Lan said that her cooking was not very good. One day, Chen Xiang visited Yu Lan’s home and tasted her cooking. The food was excellent. Chen Xiang said, “Your cooking is really not good!”

LC Chinese: 喻蓝说自己做的菜不太好吃。有天陈向来喻蓝家做客，尝到了喻蓝做的菜，味道不太好。陈向说：“你做的菜可不太好吃啊。”

English: Yu Lan said that her cooking was not very good. One day, Chen Xiang visited Yu Lan’s home and tasted her cooking. The food was not good. Chen Xiang said, “Your cooking is really not good!”

IC Chinese: 喻蓝说自己做的菜非常好吃。有天陈向来喻蓝家做客，尝到了喻蓝做的菜，味道不太好。陈向说：“你做的菜可真好吃啊。”

English: Yu Lan said that her cooking was very delicious. One day, Chen Xiang visited Yu Lan’s home and tasted her cooking. The food was not good. Chen Xiang said, “Your cooking is really delicious!”

Framework 4 – Singing

LP Chinese: 魏达告诉梅军自己唱歌很好听。他俩一起参加学校合唱团，结果魏达唱歌非常好听悦耳。梅军说：“你唱的真好听啊”。

English: Wei Da told Mei Jun that he was a good singer. They joined the school choir together, and Wei Da’s singing was very pleasant. Mei Jun said, “You sing really well!”

IP Chinese: 魏达告诉梅军自己唱歌很难听。他俩一起参加学校合唱团，结果魏达唱歌非常好听悦耳。梅军说：“你唱的真难听啊”。

English: Wei Da told Mei Jun that he was a terrible singer. They joined the school choir together, and Wei Da’s singing was very pleasant. Mei Jun said, “You sing really badly!”

LC Chinese: 魏达告诉梅军自己唱歌很难听。他俩一起参加学校合唱团，结果魏达唱歌不太好听，老走调。梅军说：“你唱的真难听啊”。

English: Wei Da told Mei Jun that he was a terrible singer. They joined the school choir together, and Wei Da’s singing was not good; he was often off-key. Mei Jun said, “You sing really badly!”

IC Chinese: 魏达告诉梅军自己唱歌很好听。他俩一起参加学校合唱团，结果魏达唱歌不太好听，老走调。梅军说：“你唱的真好听啊”。

English: Wei Da told Mei Jun that he was a good singer. They joined the school choir together, and Wei Da’s singing was not good; he was often off-key. Mei Jun said, “You sing really well!”

Framework 5 – Homework

LP Chinese: 刘浩预料这次自己的作业成绩会很好。结果作业发下来他对近满分的成绩感到欣喜。吴燕对他说：“你可真是太厉害了”。

English: Liu Hao expected that his homework grade would be very good. When the homework was returned, he was delighted to get nearly a perfect score. Wu Yan said to him, “You are really amazing!”

IP Chinese: 刘浩预料这次自己的作业成绩会很差。结果作业发下来他对勉强及格的成绩感到失望。吴燕对他说：“你可真是太厉害了”。

English: Liu Hao expected that his homework grade would be very poor. When the homework was returned, he was disappointed with a barely passing score. Wu Yan said to him, “You are really amazing!”

LC Chinese: 刘浩预料这次自己的作业成绩会很差。结果作业发下来他对近满分的成绩感到欣喜。吴燕对他说：“你可真是太糟糕了”。

English: Liu Hao expected that his homework grade would be very poor. When the homework was returned, he was delighted to get nearly a perfect score. Wu Yan said to him, “You are really terrible!”

IC Chinese: 刘浩预料这次自己的作业成绩会很好。结果作业发下来他对勉强及格的成绩感到失望。吴燕对他说：“你可真是太糟糕了”。

English: Liu Hao expected that his homework grade would be very good. When the homework was returned, he was disappointed with a barely passing score. Wu Yan said to him, “You are really terrible!”

Framework 6 – Opening a Bottle

LP Chinese: 爽爽说自己力气很大。她试图拧开一个瓶盖，一下子就打开了。李静对她说：“你可真是太强了”。

English: Shuang Shuang said she was very strong. She tried to unscrew a bottle cap and opened it immediately. Li Jing said to her, “You are really strong!”

IP Chinese: 爽爽说自己力气很小。她试图拧开一个瓶盖，一下子就打开了。李静对她说：“你可真是太弱了”。

English: Shuang Shuang said she was very weak. She tried to unscrew a bottle cap and opened it immediately. Li Jing said to her, “You are really weak!”

LC Chinese: 爽爽说自己力气很小。她试图拧开一个瓶盖，拧了半天没拧开。李静对她说：“你可真是太弱了”。

English: Shuang Shuang said she was very weak. She tried to unscrew a bottle cap but could not open it after trying for a long time. Li Jing said to her, “You are really weak!”

IC Chinese: 爽爽说自己力气很大。她试图拧开一个瓶盖，拧了半天没拧开。李静对她说：“你可真是太强了”。

English: Shuang Shuang said she was very strong. She tried to unscrew a bottle cap but could not open it after trying for a long time. Li Jing said to her, “You are really strong!”

Framework 7 – Losing Keys

LP Chinese: 大鹏说自己从不丢三落四。昨天将钥匙落在教室里马上就意识到找了回来。安妮说：“你可真是小心谨慎呀”。

English: Da Peng said he was never absent-minded. Yesterday he left his keys in the classroom but realized it immediately and retrieved them. An Ni said, “You are really careful!”

IP Chinese: 大鹏说自己经常丢三落四。昨天将钥匙落在教室里马上就意识到找了回来。安妮说：“你可真是粗心大意呀”。

English: Da Peng said he was often absent-minded. Yesterday he left his keys in the classroom but realized it immediately and retrieved them. An Ni said, “You are really careless!”

LC Chinese: 大鹏说自己经常丢三落四。昨天将钥匙落在教室第二天才意识到丢了。安妮说：“你可真是粗心大意呀”。

English: Da Peng said he was often absent-minded. Yesterday he left his keys in the classroom and only realized they were lost the next day. An Ni said, “You are really careless!”

IC Chinese: 大鹏说自己从不丢三落四。昨天将钥匙落在教室第二天才意识到丢了。安妮说：“你可真是小心谨慎呀”。

English: Da Peng said he was never absent-minded. Yesterday he left his keys in the classroom and only realized they were lost the next day. An Ni said, “You are really careful!”

Framework 8 – Exam

LP Chinese: 何宇告诉真真自己这次考试可能考的不错。成绩下来了何宇考了全班第一。真真说：“你考的也太好了吧”。

English: He Yu told Zhen Zhen that he might do well on the exam. When the results came out, He Yu ranked first in the class. Zhen Zhen said, “You did so well!”

IP Chinese: 何宇告诉真真自己这次考试可能考的不好。成绩下来了何宇考了全班第一。真真说：“你考的也太差了吧”。

English: He Yu told Zhen Zhen that he might do poorly on the exam. When the results came out, He Yu ranked first in the class. Zhen Zhen said, “You did so poorly!”

LC Chinese: 何宇告诉真真自己这次考试可能考的不好。成绩下来了何宇考了全班末尾。真真说：“你考的也太差了吧”。

English: He Yu told Zhen Zhen that he might do poorly on the exam. When the results came out, He Yu ranked last in the class. Zhen Zhen said, “You did so poorly!”

IC Chinese: 何宇告诉真真自己这次考试可能考的不错。成绩下来了何宇考了全班末尾。真真说：“你考的也太好了吧”。

English: He Yu told Zhen Zhen that he might do well on the exam. When the results came out, He Yu ranked last in the class. Zhen Zhen said, “You did so well!”

Framework 9 – Hide and Seek

LP Chinese: 祝育告诉阿乐自己捉迷藏总能找到好的藏身之处。祝育藏阿乐倒数，阿乐找半天也没有找到祝育的位置。阿乐说：“你藏的真好！”

English: Zhu Yu told A Le that she always found good hiding places in hide-and-seek. Zhu Yu hid while A Le counted, and A Le searched for a long time without finding her. A Le said, “You hid really well!”

IP Chinese: 祝育告诉阿乐自己捉迷藏总找不到好的藏身之处。祝育藏阿乐倒数，阿乐找半天也没有找到祝育的位置。阿乐说：“你太好找了！”

English: Zhu Yu told A Le that she never found good hiding places. Zhu Yu hid while A Le counted, and A Le searched for a long time without finding her. A Le said, “You are too easy to find!”

LC Chinese: 祝育告诉阿乐自己捉迷藏总找不到好的藏身之处。祝育藏阿乐倒数，阿乐一下子就找到祝育的位置。阿乐说：“你太好找了！”

English: Zhu Yu told A Le that she never found good hiding places. Zhu Yu hid while A Le counted, and A Le found her immediately. A Le said, “You are too easy to find!”

IC Chinese: 祝育告诉阿乐自己捉迷藏总能找到好的藏身之处。祝育藏阿乐倒数，阿乐一下子就找到祝育的位置。阿乐说：“你藏的真好！”

English: Zhu Yu told A Le that she always found good hiding places. Zhu Yu hid while A Le counted, and A Le found her immediately. A Le said, “You hid really well!”

Framework 10 – Basketball

LP Chinese: 王栋告诉华兰自己打篮球很棒。他们一起比赛到了最后一分钟，王栋投篮投中得分。华兰说：“这真是个好球啊！”

English: Wang Dong told Hua Lan that he was a great basketball player. They played a game, and in the final minute, Wang Dong made a shot and scored. Hua Lan said, “That was a great shot!”

IP Chinese: 王栋告诉华兰自己打篮球很烂。他们一起比赛到了最后一分钟，王栋投篮投中得分。华兰说：“刚才那球打的太烂了”。

English: Wang Dong told Hua Lan that he was a terrible basketball player. They played a game, and in the final minute, Wang Dong made a shot and scored. Hua Lan said, “That shot was so bad!”

LC Chinese: 王栋告诉华兰自己打篮球很烂。他们一起比赛到了最后一分钟，王栋投篮投偏未得分。华兰说：“刚才那球打的太烂了”。

English: Wang Dong told Hua Lan that he was a terrible basketball player. They played a game, and in the final minute, Wang Dong missed the shot. Hua Lan said, “That shot was so bad!”

IC Chinese: 王栋告诉华兰自己打篮球很棒。他们一起比赛到了最后一分钟，王栋投篮投偏未得分。华兰说："这真是个好球啊！"

English: Wang Dong told Hua Lan that he was a great basketball player. They played a game, and in the final minute, Wang Dong missed the shot. Hua Lan said, "That was a great shot!"

Framework 11 – Disneyland

LP Chinese: 小梦说今天人少去迪士尼不会排队。结果小梦和小文去迪士尼，里面所有的项目都不需要排队。小文说："今天真是来迪士尼的好时候！"

English: Xiao Meng said that because there were few people, there would be no queues at Disneyland. When they went, none of the rides required waiting. Xiao Wen said, "This is really a great time to come to Disneyland!"

IP Chinese: 小梦说今天人多去迪士尼会排队。结果小梦和小文去迪士尼，里面所有的项目都不需要排队。小文说："今天真不是来迪士尼的好时候！"

English: Xiao Meng said that because there were many people, there would be queues at Disneyland. When they went, none of the rides required waiting. Xiao Wen said, "This is really not a good time to come to Disneyland!"

LC Chinese: 小梦说今天人多去迪士尼会排队。结果小梦和小文去迪士尼，里面所有的项目都需要排队。小文说："今天真不是来迪士尼的好时候！"

English: Xiao Meng said that because there were many people, there would be queues at Disneyland. When they went, all the rides required waiting. Xiao Wen said, "This is really not a good time to come to Disneyland!"

IC Chinese: 小梦说今天人少去迪士尼不会排队。结果小梦和小文去迪士尼，里面所有的项目都需要排队。小文说："今天真是来迪士尼的好时候！"

English: Xiao Meng said that because there were few people, there would be no queues at Disneyland. When they went, all the rides required waiting. Xiao Wen said, "This is really a great time to come to Disneyland!"

Framework 12 – Kite

LP Chinese: 小阳说自己做的风筝可能飞得很高。小妍想要试飞小阳做的风筝，风筝飞了一会很快就飞得很高。小妍说："你做的风筝果然很好啊"。

English: Xiao Yang said that his homemade kite might fly very high. Xiao Yan tried to fly it, and after a while it quickly went up high. Xiao Yan said, "Your kite is really good!"

IP Chinese: 小阳说自己做的风筝可能飞不高。小妍想要试飞小阳做的风筝，风筝飞了一会很快就飞得很高。小妍说："你做的风筝果然不是很好啊"。

English: Xiao Yang said that his homemade kite might not fly high. Xiao Yan tried to fly it, and after a while it quickly went up high. Xiao Yan said, "Your kite is really not very good!"

LC Chinese: 小阳说自己做的风筝可能飞不高。小妍想要试飞小阳做的风筝，风筝飞了一会很快落地了。小妍说："你做的风筝果然不是很好啊"。

English: Xiao Yang said that his homemade kite might not fly high. Xiao Yan tried to fly it, and after a while it quickly fell to the ground. Xiao Yan said, "Your kite is really not very good!"

IC Chinese: 小阳说自己做的风筝可能飞得很高。小妍想要试飞小阳做的风筝，风筝飞了一会很快落地了。小妍说："你做的风筝果然很好啊"。

English: Xiao Yang said that his homemade kite might fly very high. Xiao Yan tried to fly it, and after a while it quickly fell to the ground. Xiao Yan said, "Your kite is really good!"

Appendix S2. Pretest Results

A pretest was conducted with 125 third-grade students. Participants rated difficulty on a 5-point scale and judged whether each item was ironic. Items with inconsistent irony identification or inappropriate difficulty were excluded. The final 12 story frameworks showed high inter-rater agreement (Cohen's κ = 0.85).

Story	Difficulty Mean	SD	Irony identification (%)
1	1.42	0.56	92.8
2	1.51	0.61	90.4
3	1.25	0.48	94.4
4	1.47	0.55	90.7
5	1.55	0.49	92.0
6	1.46	0.60	89.8
7	1.52	0.57	91.3

8	1.22	0.48	92.9
9	1.29	0.52	93.5
10	1.31	0.57	94.1
11	1.36	0.72	92.5
12	1.44	0.74	91.5

Appendix S3. Timeflow of a trial (how screen switched)

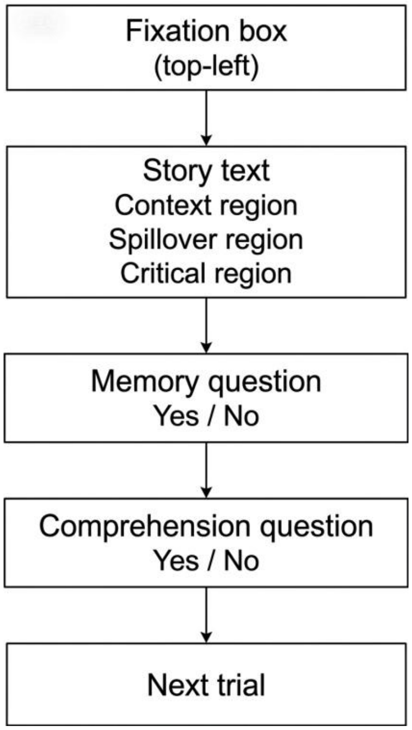

Supplement: Supplementary file 1 [file behavsci-16-01101-s001.zip › behavsci-4311182-supplementary2.pdf]
